# Pharmacy-based hypertension care employing mHealth in Lagos, Nigeria – a mixed methods feasibility study

**DOI:** 10.1186/s12913-018-3740-3

**Published:** 2018-12-04

**Authors:** Heleen E. Nelissen, Anne L. Cremers, Tochi J. Okwor, Sam Kool, Frank van Leth, Lizzy Brewster, Olalekan Makinde, René Gerrets, Marleen E. Hendriks, Constance Schultsz, Akin Osibogun, Anja H. van’t Hoog

**Affiliations:** 1 Department of Global Health, Amsterdam UMC, University of Amsterdam, Meibergdreef 9, Amsterdam, the Netherlands; 2 Amsterdam Institute for Global Health and Development, Paasheuvelweg 25, Amsterdam, The Netherlands; 3 Department of Anthropology, University of Amsterdam, Nieuwe Achtergracht 166, Amsterdam, The Netherlands; 4 Department of Infectious Diseases, Division of Internal Medicine, Amsterdam UMC, University of Amsterdam, Center of Tropical Medicine and Travel Medicine, Meibergdreef 9, Amsterdam, The Netherlands; 5 Centre for Epidemiology and Health Development, Ibeju, Lekki, Lagos Nigeria; 6 Department of Community Health, University of Nigeria Teaching Hospital Enugu, P.M.B, Enugu, 01129 Nigeria; 7 Department of Community Health, Lagos University Teaching Hospital, Idi-Araba, Lagos, Nigeria; 8 Joep Lange Institute, Paasheuvelweg 25, Amsterdam, The Netherlands

**Keywords:** Hypertension, Pharmacy care, Decentralization, Task-shifting, mHealth, Private sector, Quality of care, Health services research, Feasibility, Sub-Saharan Africa

## Abstract

**Background:**

Access to quality hypertension care is often poor in sub-Saharan Africa. Some community pharmacies offer hypertension monitoring services, with and without involvement of medical doctors. To directly connect pharmacy staff and cardiologists a care model including a mobile application (mHealth) for remote patient monitoring was implemented and pilot tested in Lagos, Nigeria. Pharmacists provided blood pressure measurements and counselling. Cardiologists enrolled patients in the pilot program and remotely monitored them, for which patients paid a monthly fee. We evaluated the feasibility of this care model at five private community pharmacies. Outcome measures were retention in care, blood pressure change, quality of care, and patients’ and healthcare providers’ satisfaction with the care model.

**Methods:**

Patients participated in the care model’s pilot at one of the five pharmacies for approximately 6–8 months from February 2016. We conducted structured patient interviews and blood pressure measurements at pilot entry and exit, and used exports of the mHealth-application, in-depth interviews and focus group discussions with patients, pharmacists and cardiologists.

**Results:**

Of 336 enrolled patients, 236 (72%) were interviewed at pilot entry and exit. According to the mHealth data 71% returned to the pharmacy after enrollment, with 3.3 months (IQR: 2.2–5.4) median duration of activity in the mHealth-application. Patients self-reported more visits than recorded in the mHealth data. Pharmacists mentioned use of paper records, understaffing, the application not being user-friendly, and patients’ unwillingness to pay as reasons for underreporting. Mean systolic blood pressure decreased 9.9 mmHg (SD: 18). Blood pressure on target increased from 24 to 56% and an additional 10% had an improved blood pressure at endline, however this was not associated with duration of mHealth activity. Patients were satisfied because of accessibility, attention, adherence and information provision.

**Conclusion:**

Patients, pharmacists and cardiologists adopted the care model, albeit with gaps in mHealth data. Most patients were satisfied, and their mean blood pressure significantly reduced. Usage of the mHealth application, pharmacy incentives, and a modified financing model are opportunities for improvement. In addition, costs of implementation and availability of involved healthcare providers need to be investigated before such a care model can be further implemented.

**Electronic supplementary material:**

The online version of this article (10.1186/s12913-018-3740-3) contains supplementary material, which is available to authorized users.

## Background

In sub-Sahara Africa (SSA), the awareness of hypertension and the coverage of antihypertensive treatment is low, mainly due to poor access to quality care [1]. Accessibility to care for patients is often low because of high costs of care, high travel costs, or long waiting times at clinics, leading to loss of income and low patient satisfaction [2]. Nevertheless, blood pressure reduction through treatment greatly reduces the risk of cardiovascular disease (CVD).

In Lagos, Nigeria, patients often seek care outside the formal healthcare system [3]. Hypertension care services of varying quality are offered by a wide range of formal and informal healthcare providers. Patients often seek healthcare in their community, for example at community pharmacies, patent and proprietary medicine vendors, or traditional healers [4]. Types of services and procedures available at community pharmacies differ and are mostly not standardized according to national guidelines, and medical doctors are not necessarily involved. Systematic reviews on pharmacy-based care conducted in Asia, Australia, Europe, and North- and South-America, concluded that pharmacist interventions can improve blood pressure management [5, 6]. However, data from African settings is limited, especially from interventions that routinely involve medical doctors. Three studies from Nigeria [7–9] found blood pressure reductions with pharmacy-based care which involved medical doctors if the need arose. In addition, several studies from SSA describe successful CVD prevention programs led by non-medical doctors [10–17].

Community pharmacies play an important role in the Nigerian healthcare system [18] and task shifting to pharmacists may be an opportunity to deliver healthcare considering a shortage of medical doctors. Nigeria has less than 4 doctors per 10.000 people [19], which is far below the World Health Organization (WHO) guideline of 23 doctors per 10.000 people [19], making quality of care a concern. Previous studies from Nigeria found that most pharmacists, in addition to dispensing, also prescribe medications [20, 21]. In these studies, medical doctors were generally reluctant to expanding the activities of the pharmacist. They acknowledge the potential to improve access to treatment and reduce waiting times, but see prescribing as their responsibility and doubt pharmacists’ skills to make an adequate diagnosis [20]. Patients [20, 22], as well as 50% of the pharmacists [21], preferred prescriptions through a collaboration between medical doctors and pharmacists, to ensure a correct diagnosis. Currently, there is no formal system in place to foster collaborative working relationships between medical doctors and community pharmacists in Nigeria [21].

Medical and public health practice supported by mobile devices (mHealth) is a potential way to improve communication between medical doctors and pharmacists. Simultaneously, patient health outcomes can be monitored, access to care improved, and waiting time and medical doctors’ workload reduced [23]. Tele-monitoring, i.e. remote patient monitoring, is also an essential component of a successful pharmacy-based hypertension program in the United States [24]. Studies from other high-income countries show that tele-monitoring of blood pressure was associated with blood pressure reductions [25]. The WHO supports the use of mHealth strategies to combat non-communicable diseases [26].

Pharmacy-based hypertension care that includes remote patient monitoring by cardiologists through mHealth may be an effective way to improve access to hypertension care and blood pressure control and subsequently reduce CVD in SSA. To assess the feasibility of such an approach, a pharmacy-based hypertension care model employing mHealth was piloted in Lagos, Nigeria, for six months and evaluated in a mixed-methods study. Here we report on the following study outcomes: 1) patient retention in the pilot program and reasons for dropping-out, 2) changes in blood pressure during the pilot program and determinants for blood pressure on target or improvement, and 3) the quality of and satisfaction with pharmacy-based care including mHealth. Patients and healthcare providers’ perceptions and practices regarding hypertension, pharmacy-based care, and mHealth [18], and our experiences with recruitment of patients in the pilot program [27] are presented elsewhere.

## Methods

### Pharmacy-based care model

Providing care through private community pharmacies was recognized by OMRON Healthcare Europe (“OMRON”) as a potential model to increase access to and quality of hypertension care in Lagos, Nigeria. The key component of the model was task-shifting from medical doctors to pharmacy staff by using a mobile application (“mHealth app”), developed jointly by OMRON and technical partner Orange. OMRON implemented the care model, including the mHealth app, with support from PharmAccess Nigeria. Five private community pharmacies serving low- and/or middle-income communities were selected from OMRON’s pool of retail outlets, based on the pharmacist’ interest to participate in the pilot program and their spread over Lagos State. The included pharmacies were officially registered, and the main pharmacist received at least five years of professional training. Cardiologists and pharmacists were additionally trained by OMRON in their roles and responsibilities in the pilot program (see Additional file 1), the mHealth app, patient education, counselling, clinical guidelines, lifestyle measures, the blood pressure device, and data confidentiality.

Patients were identified and recruited in the pilot program through community hypertension screening events, the participating pharmacies, and three outpatient clinics of Lagos University Teaching Hospital (LUTH) from February to May 2016 [27]. At recruitment, three resident cardiologists and two general practitioners under supervision of a cardiologist assessed inclusion and exclusion criteria (listed below), ineligible individuals were referred to regular hypertension care if necessary. Included patients were registered in the pilot program and mHealth app and anticipated to stay in the pilot program for approximately six months. From October to December 2016 patients were invited for an exit consultation and referral to regular care. The patient participation fee was 250 Naira per month (≈0.96USD, average exchange rate May-Dec 2016), excluding the costs of medications. After recruitment, the three cardiologists were responsible for remote patient monitoring and management, together with the pharmacists, and communication between them was primarily through the mHealth app. The face-to-face interaction between the cardiologist and patient was limited to the recruitment visit and visits requested by the cardiologist if needed based on the mHealth records. The pharmacist and patient interacted with each other at the pharmacy. The role of the pharmacy staff was to perform regular consultations with the patients (including blood pressure measurements and medication- and lifestyle counselling), to remind patients of their consultation and outstanding prescriptions, and to communicate with the cardiologist on concerns regarding the patients’ health. Cardiologists and pharmacists received a fee for each patient monitored, irrespective of the number of prescriptions or visits.

#### Inclusion and exclusion criteria

The criteria for inclusion in the pilot program included individuals aged 18 years and above and a (new or previous) hypertension diagnosis confirmed by the cardiologist or general practitioner. Exclusion criteria were: 1) individuals with a previous history of cardiac failure, stroke or renal disease; additional risk factors for CVD identified by the cardiologist or general practitioner; individuals with a systolic blood pressure (SBP) ≥ 180 mmHg and/or diastolic blood pressure (DBP) ≥ 110 mmHg were not suitable for the pilot program, as more comprehensive monitoring may be desired, which could not be guaranteed during this pilot phase; 2) individuals not permanently residing in Lagos State; and 3) pregnant women (self-reported).

### Feasibility study

#### Study design and data collection

**Fig. 1:**
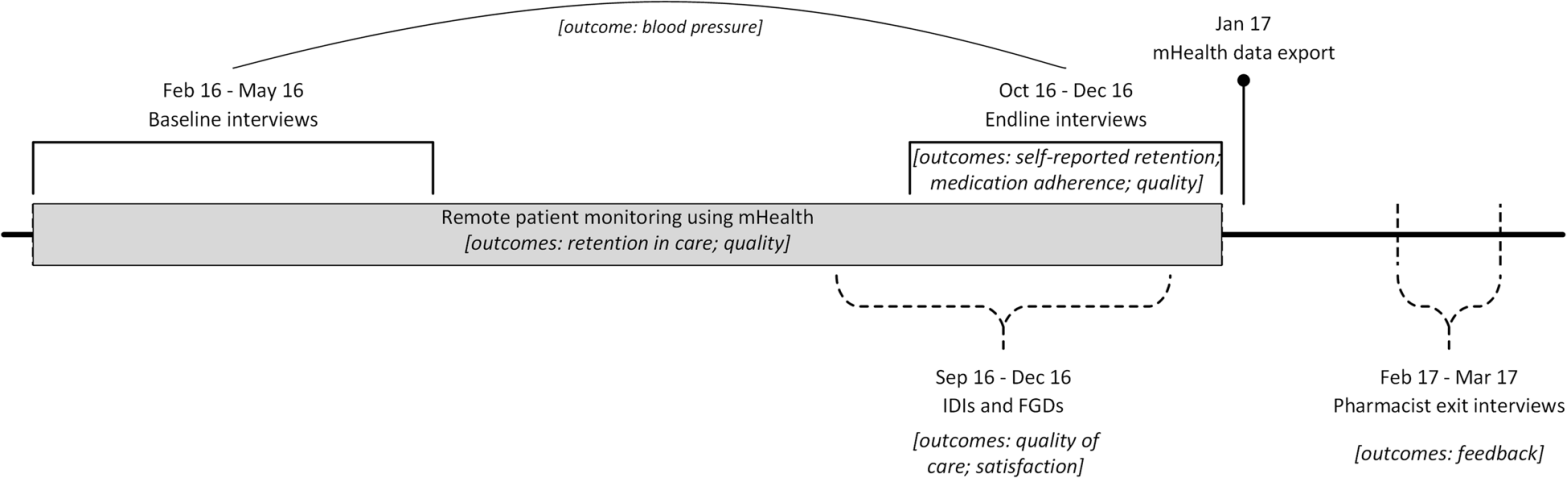
Feasibility study timeline including study outcomes

We used a mixed-methods approach to evaluate the feasibility of the pilot program. Patients participating in the pilot program were also required to participate in the baseline and endline interviews of the feasibility study. To measure retention in care the minimum required sample size was estimated at 300 patients, but we aimed to include 500 patients (around 83 patients per pharmacy and 83 patients from LUTH; see Additional file 1). The feasibility study was designed, conducted and supervised by an independent research team. Data was collected between February 2016 and March 2017 by research assistants from Lagos trained and supervised by researchers from Lagos and Amsterdam. The following data sources were used (visualized in the timeline in Fig. 1):

1. Baseline interviews were conducted with patients after recruitment in the pilot program. The interview contained structured questions on patient demographics, healthcare seeking behavior, medication and lifestyle adherence, prescribed medication, anthropometric measurements, and blood pressure measurements.
2. Endline interviews were conducted during the exit consultations. The interview contained structured questions on healthcare seeking behavior during the pilot program and experiences with the pilot program, medication and lifestyle adherence, side effects and complaints, anthropometric measurements, and blood pressure measurements. Reasons for loss to follow-up were recorded for patients who did not show up or could not be reached. Baseline and endline interview data were collected using ODK Collect [28]. For quality control, all data was reviewed by the local supervisor before uploading to the server, and queries were sent to the local supervisor for data quality checks.
3. mHealth data: an export of digital patient records completed in the mHealth app including routine patient data entered by pharmacy staff and cardiologists, containing information on pharmacy and doctor consultations, blood pressure measurements, and prescribed and dispensed medication. Data was recorded from the start of the pilot program for at least six months per patient. The last activity in the mHealth records was observed November 19th, 2016 and the data was exported January 3rd, 2017. Data was de-identified and prepared for analysis by creating a timeline of events for each patient using custom software. The research team did not monitor nor otherwise influence the recording of this data.

For our outcome related to the quality of, and satisfaction with the pharmacy-based care pilot program, we selected data on these themes obtained in the qualitative component of the study. We used the following methods, which are described elsewhere in more detail [18]:

4. In-depth interviews (IDIs) with 15 patients, five pharmacists, and three cardiologists participating in the pilot program.
5. Focus group discussions (FGDs) with five patients (*n* = 2) and with five pharmacists (*n* = 1) participating in the pilot program.
6. Pharmacist exit interviews were conducted with each pharmacist (*n* = 5) after completion of the pilot program. The interview guide was created for each pharmacy based upon a first analysis of the qualitative data, and a preliminary data report on retention in care in the mHealth data and patients’ self-report at endline.

#### Statistical analysis and outcome definitions

The mHealth data, and the baseline and endline interview data were analyzed using Stata version 12 (StataCorp LP, College Station, Texas, USA). We here describe the definitions of our study outcomes, data sources (Fig. 1) and statistical analyses.

##### Retention in care

By using the prospective mHealth data of our cohort we analyzed whether patients had activity, defined as any recording of patient data in the mHealth app after registration, or if not registered, the date of the baseline interview. Among patients with activity in the mHealth app, we calculated the duration of activity between registration and the last recorded visit at the pharmacy or cardiologist, and the number of pharmacy visits made during this period. Differences between groups were tested using the Chi^2^-test and the Wilcoxon-Mann-Whitney test. Patient’s self-reported number of pharmacy visits was obtained during the endline interviews. Self-reported retention in care was defined as six or more pharmacy visits during the pilot program, including the recruitment visit. This definition was used as medical doctors in Nigeria generally prescribe antihypertensive medication for one month and the duration of the pilot program was anticipated for six months per patient.

##### Blood pressure

We made a before-after comparison using the baseline and endline measurements to calculate differences in mean SBP, DBP, and blood pressure on target and/or an improved blood pressure at endline. Blood pressure was measured three times on the upper left arm at heart level after at least 5 min of rest in a sitting position using a validated automatic blood pressure device (OMRON M6 Comfort; OMRON Corporation, Kyoto, Japan). The mean value of the second and third measurement was used for analysis. Blood pressure on target was defined as a SBP < 140 mmHg and DBP < 90 mmHg in patients aged below 60 years and those with self-reported diabetes mellitus, and for patients above 60 years SBP < 150 mmHg and DBP < 90 mmHg [29, 30]. Improved blood pressure was defined as a ≥ 10% decrease in SBP or DBP, ≥ 20 mmHg decrease in SBP, or ≥ 10 mmHg decrease in DBP. Change in blood pressure between the baseline and endline interview was compared by a paired *t*-test and change in blood pressure between groups was compared by an independent *t*-test.

##### Medication adherence

The baseline and endline interviews included the 8-item Morisky Medication Adherence Scale (MMAS-8) to assess self-reported adherence to antihypertensive medication. A score below 6 was classified as low adherence, a score between 6 and 8 moderate adherence and a score of 8 high adherence [31]. Self-reported adherence to lifestyle advice was assessed on a 5-point Likert scale (1–5). If the patient received multiple lifestyle advices (i.e. on diet, alcohol use, smoking cessation, physical activity) the average response on the Likert scale was used. Low adherence to lifestyle advice was define as a score from 0 to 3, moderate adherence from 3 to 4 and high adherence above 4.

##### Quality of care

The cardiologist’s lag time was calculated using the prospective mHealth data of our cohort, defined as the duration between a recorded blood pressure measurement by the pharmacy and the cardiologist’s response in the mHealth app. Side effects and complaints, and satisfaction with the pilot program were summarized from the endline interviews.

##### Regression analyses

We further investigated which factors contributed to changes in blood pressure and hypothesized that if the mHealth app provided a good reflection of the care provided, longer duration of activity in the mHealth app would be associated with an increased probability of blood pressure on target and/or improved blood pressure at the endline interview. We constructed a multilevel logistic regression model of patients nested within pharmacies with duration active in the mHealth app as primary exposure variable. We additionally added patient characteristics (gender, age, newly diagnosis, antihypertensive medication use, entry into the pilot program) and risk factors for hypertension that may influence patient retention (body mass index, self-reported diabetes mellitus, smoking status, alcohol use, adherence to antihypertensive medication, adherence to lifestyle advice) and blood pressure to the model regardless of statistical significance. In addition, household wealth, highest completed grade of education and plausible interactions between duration of activity in the mHealth app and co-variates were explored and maintained based on the likelihood ratio test (*p*-value ≤0.05).

We performed three additional analyses: a sensitivity analysis was conducted to assess the robustness of the results when applying a stricter outcome definition using of blood pressure on target. A second sensitivity analysis was conducted to assess potential bias from inconsistent use of the mHealth app among pharmacies. This analysis excluded patients without activity in the mHealth app beyond enrollment. Lastly, to examine if patient’s self-reported retention would reflect provided care in the mHealth data, we replicated the multilevel logistic regression model obtained in the first analysis, and substituted duration of activity in the mHealth app with patient’s self-reported retention. We then assessed if the size of the effect measure and its significance was different compared to first analysis.

#### Qualitative analysis

Research assistants transcribed IDIs and FGDs and thematic content analysis was performed using Dedoose Version 7.0.23. Data was blindly double-coded, and content analyzed for meaning and patterns using grounded theory [18].

## Results

### Patient population

**Fig. 2:**
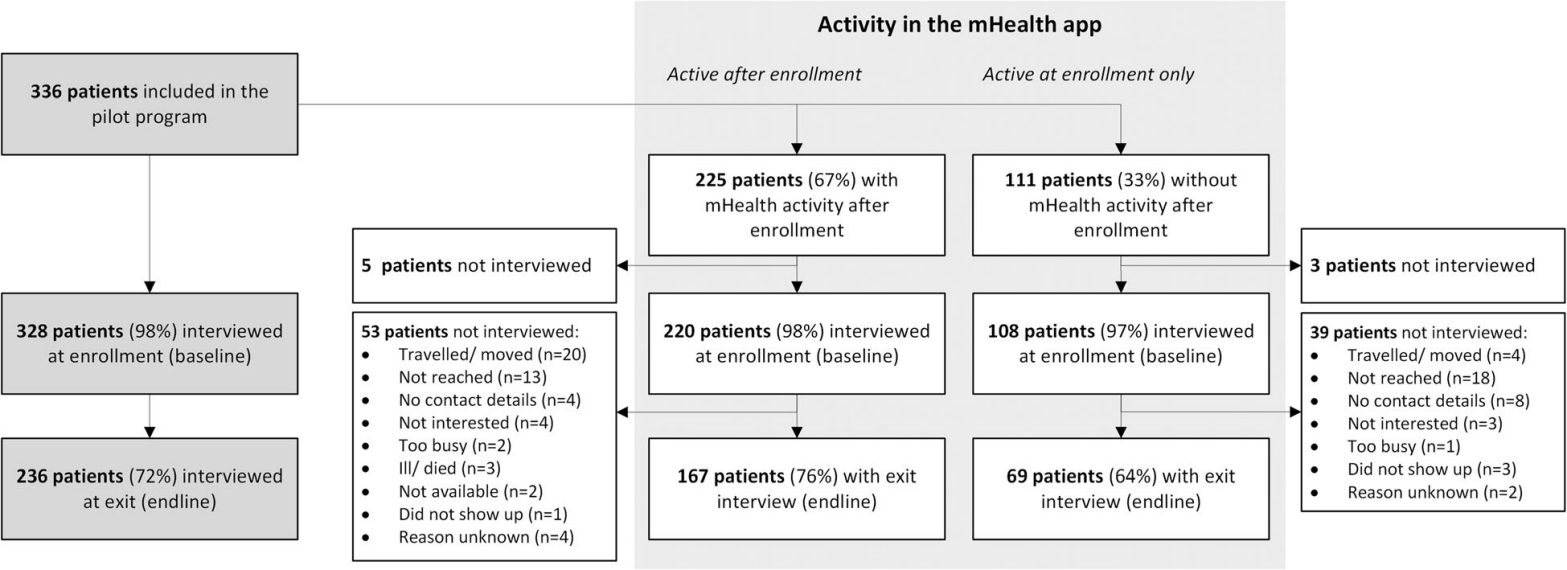
Flowchart of the population enrolled in the pilot program and feasibility study

In total 336 adults with a confirmed diagnosis of hypertension were registered in the pilot program and mHealth app. The number of included patients varied per pharmacy, from 38 to 117. A baseline interview was available for 328 patients (98%), their characteristics are shown in Table 1. At pilot exit, 236 patients (72%) had and endline interview (Fig. 2). The median duration between the baseline and endline interview was 7.1 months (inter quartile range [IQR]: 6.4–8.1 months, min.: 4.5 max.: 9.9).

**Table 1:** Baseline characteristics of patients in the feasibility study (*N* = 328)

	All (N = 328)	
	*n/ mean*	*%/ SD*
Gender, n (%)		
Male	135	(41.2)
Female	193	(58.8)
Age, mean (SD)	54.9	(11.9)
Highest degree in school completed, n (%)		
No school at all	37	(11.3)
Primary	66	(20.1)
Secondary	115	(35.1)
Tertiary	110	(33.5)
Systolic BP, mean (SD)	147.8	(16.4)
Diastolic BP, mean (SD)	90.9	(11.4)
BP classification, n (%)		
Pre-hypertensive (BP 120–139/80–89)	2	(0.6)
Stage 1 HT (BP 140–159/90–99)	142	(43.3)
Stage 2 HT (BP ≥160/100)	107	(32.6)
BP on target	77	(23.5)
Newly diagnosed, n (%)	65	(19.8)
On antihypertensive medication, n (%)	212	(64.6)
Entry into the pilot program, n (%)		
Via community screening	100	(30.5)
Via pharmacy	226	(68.9)
Via LUTH	2	(0.6)
BMI, mean (SD)	28.6	(6.1)
Self-reported DM, n (%)	29	(8.8)
Smoking status, n (%)		
Not smoking	285	(86.9)
Quitted	36	(11)
Smokes	7	(2.1)
Any alcohol use, n (%)	97	(29.6)

*BP* blood pressure, *HT* hypertension, *LUTH* Lagos University Teaching Hospital, *BMI* body mass index, *DM* diabetes mellitus

### Retention in care

Of the 336 patients in the pilot program, the median duration of activity was 2.1 months (IQR: 0.0–3.9) and a median of 3 pharmacy visits (IQR: 1–6) was recorded by the pharmacy staff. Of these patients 111 (33%) did not have any activity in the mHealth app after enrollment. Of the 236 patients with completed interviews at baseline and endline, mHealth activity was present for 71% (*n* = 165), ranging from 38 to 83% across pharmacies (Table 2). Overall among patients with mHealth activity the median duration of activity was 3.3 months (IQR: 2.2–5.4) and a median of five pharmacy visits (IQR: 3–6) was recorded by the pharmacy staff. The median number of visits across pharmacies ranged from two to six. The duration of mHealth activity did not differ between patients enrolled through community screening (4.2 months, IQR: 0.7–5.8) or through the pharmacies (3.0 months, IQR: 2.2–4.6, *p*-value = 0.645).

**Table 2:** Descriptive statistics of primary outcomes for patients with an endline interview (*N* = 232)^a^

	N	Pharmacy 1		N	Pharmacy 2		N	Pharmacy 3		N	Pharmacy 4		N	Pharmacy 5		N	ALL	
		*n/ median/ mean*	*%/ IQR/ SD*		*n/ median/ mean*	*%/ IQR/ SD*		*n/ median/ mean*	*%/ IQR/ SD*		*n/ median/ mean*	*%/ IQR/ SD*		*n/ median/ mean*	*%/ IQR/ SD*		*n/ median/ mean*	*%/ IQR/ SD*
Patients with endline interview^a^		51			42			32			83			24			232	
Retention in care																		
Activity in mHealth data after enrollment, n (%)	51	41	(80.4)	42	35	(83.3)	32	13	(40.6)	83	67	(80.7)	24	9	(37.5)	232	165	(71.1)
If active: Duration of activity in mHealth data in months, median (IQR)	41	3.9	(1–5.4)	35	4.2	(2–5.8)	13	3.2	(2–5)	67	3.2	(2.3–5.4)	9	2.7	(0.3–4.6)	165	3.3	(2.2–5.4)
If active: No. of pharmacy visits in mHealth data, median (IQR)	41	3	(2–6)	35	5	(3–9)	13	4	(2–4)	67	6	(5–7)	9	2	(2–2)	165	5	(3–6)
If active: No. of self-reported pharmacy visits, median (IQR)^b^	40	6	(4–7)	34	7	(5–13)	13	10	(7–13)	65	7	(5–13)	9	3	(2–4)	161	6	(4–11)
If active: Ratio pharmacy visits mHealth data vs. self-reported, median (IQR)	40	1.5	(1.1–2.5)	34	1.4	(1–2)	13	3.3	(2.5–4.3)	65	1.3	(0.8–1.8)	9	1.3	(1–1.5)	161	1.5	(1–2.2)
If *not* active: Any self-reported pharmacy visits, n (%)	8	7	(87.5)	7	7	(100.0)	19	17	(89.5)	12	11	(91.7)	15	9	(60.0)	61	51	(83.6)
If *not* active: No. of self-reported pharmacy visits, median (IQR)^c^	8	5	(3.5–5.5)	7	5	(4–7)	19	6	(4–9)	12	5.5	(3–11.5)	15	2	(1–4)	61	5	(3–7)
Self-reported at least 6 pharmacy visitsd, n (%)	48	27	(56.3)	41	26	(63.4)	32	22	(68.8)	77	49	(63.6)	24	2	(8.3)	222	126	(56.8)
Change in BP																		
Baseline SBP, mean (SD)	51	142.6	(15.9)	42	146.5	(16.7)	32	145.3	(10.3)	83	151.3	(15.1)	24	147.2	(13.9)	232	147.3	(15.2)
Change in SBP, mean (SD)	51	−11.1*	(16.3)	42	−8.4*	(17.9)	32	−11.8*	(17.9)	83	−10.5*	(19.5)	24	−5.5	(17.3)	232	−9.9*	(18)
Baseline DBP, mean (SD)	51	89.3	(11)	42	88.3	(12.8)	32	89.4	(9.5)	83	93.4	(11.1)	24	90	(10.6)	232	90.6	(11.2)
Change in DBP, mean (SD)	51	−5.2*	(10.9)	42	−4.2*	(10.1)	32	−6.5*	(11.5)	83	−8.0*	(12)	24	−2.0	(11.5)	232	−5.9*	(11.4)
BP on target at baseline, n (%)	51	15	(29.4)	42	16	(38.1)	32	5	(15.6)	83	18	(21.7)	24	2	(8.3)	232	56	(24.1)
BP on target at endline, n (%)	51	29	(56.9)	42	25**	(59.5)	32	20	(62.5)	83	45	(54.2)	24	10	(41.7)	232	129**	(55.6)
BP on target and/or improved at endline, n (%)	51	34	(66.7)	42	28	(66.7)	32	21	(65.6)	83	59	(71.1)	24	11	(45.8)	232	153	(65.9)

^a^4 patients are excluded from this table, 2 patients with a BP < 140&90 at baseline who did not report use of antihypertensive medication and 2 patients who were referred from LUTH to the pilot program

^b^4 patients did not know the number of visits they made to the pharmacy and are excluded (*N* = 161)

^c^6 patients did not know the number of visits they made to the pharmacy and are excluded (*N* = 61)

^d^10 patients did not know the number of visits they made to the pharmacy and are excluded (*N* = 222)

^*^p < 0.05 in one-sample *t*-test on change between baseline and endline

**p < 0.05 in Fisher’s exact-test on the difference between BP on target at baseline and endline

*SBP* systolic blood pressure, *DBP* diastolic blood pressure, *BP* blood pressure

At the endline interview, patients self-reported a median of six pharmacy visits (IQR: 4–11) during the pilot program. Across pharmacies the median number of self-reported pharmacy visits ranged from three to 10. Patients self-reported a median of 1.5 visits (IQR: 1–2.2) more compared to the mHealth data. Of the patients without mHealth activity, 84% self-reported pharmacy visits. These patients reported a median of five visits (IQR: 3–7), significantly less than those with mHealth activity, who reported a median of 6 visits (IQR: 4–11; Kruskal Wallis-test, *p-value* < 0.001).

### Change in blood pressure and associations with retention in care

Mean blood pressure at baseline was 147.3 mmHg systolic and 90.6 mmHg diastolic, and 24.1% of the patients had their blood pressure on target (Table 2). The average change in SBP was  -9.9 mmHg (SD: 18.0) and DBP -5.9 mmHg (SD: 11.4) between baseline and endline. No difference was observed in the average change in SBP among those newly diagnosed (-9.6 mmHg, SD: 14.3) and previously diagnosed (-10.0 mmHg, SD: 18.8, *p*-value = 0.907), or those not on antihypertensive medication (-12.8 mmHg, SD: 17.8) and using antihypertensive medication at baseline (-8.5 mmHg, SD: 18.0, *p*-value = 0.083). Stratified by pharmacy, a statistically significant decrease in blood pressure was observed in 4 out of 5 pharmacies (Table 2). Blood pressure on target increased from 24% at baseline to 56% at endline (*p*-value < 0.001), and an increase was observed among all pharmacies (Table 2). An additional 10% of patients had an improved blood pressure at endline.

Both in crude and regression analysis blood pressure on target and/or an improved blood pressure was not associated with the patient’s duration of activity in the mHealth data (Table 3), suggesting possible underreporting in the mHealth app. Factors associated with blood pressure on target and/or an improved blood pressure were high self-reported adherence to medication, stage 1 hypertension at baseline, being female, increasing age, having quit smoking and higher education, while previous hypertension diagnosis, being on antihypertensive medication at baseline, entry into the pilot program, self-reported diabetes mellitus, any alcohol use, and adherence to lifestyle advice were not. Sensitivity analyses to test the robustness of the model did not change the results significantly (see Additional file 2). The self-reported number of pharmacy visits better predict blood pressure on target and/or improved blood pressure (OR = 1.55 95%CI: 0.80–3.02), although the association was likewise not significant (see Additional file 3).

**Table 3:** Multilevel logistic regression analysis on BP on target and/or improved at endline (*N* = 226)^a^

	BP on target or improved		Univariate analysis			Multivariate analysis		
	n	(%)	OR	*p*-value	95% CI	OR	*p*-value	95% CI
Duration active in mHealth app (per month)			1.03	0.610	(0.92–1.15)	1.02	0.724	(0.90–1.16)
Gender								
Male	60	58.8	ref.	–	–	ref.	–	–
Female	88	71.0	1.71	0.057	(0.98–2.97)	2.77	0.009	(1.30–5.93)
Age at baseline			1.03	0.013	(1.01–1.05)	1.04	0.017	(1.01–1.07)
BP classification at baseline								
Stage 1 HT (BP 140–159/90–99)	63	57.3	0.31	0.005	(0.14–0.70)	0.39	0.043	(0.15–0.97)
Stage 2 HT (BP ≥160/100)	46	67.6	0.62	0.107	(0.20–1.17)	0.62	0.344	(0.23–1.67)
Stage 1 HT (BP 140–159/90–99)	39	81.3	ref.	–	–	ref.	–	–
Newly diagnosed								
No	125	66.8	ref.	–	–	ref.	–	–
Yes	23	59.0	0.71	0.348	(0.35–1.45)	0.88	0.810	(0.32–2.45)
On antihypertensive medication at baseline								
No	46	62.2	ref.	–	–	ref.	–	–
Yes	102	67.1	1.24	0.464	(0.70–2.22)	0.59	0.248	(0.24–1.45)
Entry into the pilot program								
Via community screening	42	63.6	ref.	–	–	ref.	–	–
Via the pharmacy	106	66.3	1.12	0.707	(0.62–2.04)	1.12	0.738	(0.57–2.19)
BMI at baseline			1.01	0.689	(0.96–1.06)	0.99	0.717	(0.94–1.04)
Self-reported DM								
No	136	65.4	ref.	–	–	ref.	–	–
Yes	12	66.7	1.06	0.913	(0.38–2.94)	0.94	0.923	(0.30–2.97)
Smoking status at baseline								
Not smoking	128	64.6	ref.	–	–	ref.	–	–
Quitted	18	75.0	1.64	0.317	(0.62–4.32)	3.55	0.038	(1.07–11.74)
Smokes	2	50.0	0.55	0.551	(0.06–3.97)	0.88	0.912	(0.09–8.45)
Any alcohol use at baseline								
No	124	67.0	ref.	–	–	ref.	–	–
Yes	24	58.5	0.69	0.302	(0.35–1.39)	0.68	0.405	(0.28–1.67)
Adherence to antihypertensive medication at endline								
Low adherence	69	58.5	ref.	–	–	ref.	–	–
Moderate adherence	39	70.9	1.73	0.118	(0.87–3.44)	1.57	0.259	(0.72–3.44)
High adherence	40	75.5	2.19	0.035	(1.06–4.51)	2.27	0.049	(1.00–5.15)
Adherence to lifestyle advice at endline								
No lifestyle advice given	59	67.8	1.45	0.414	(0.60–3.53)	1.29	0.623	(0.47–3.55)
Low adherence	16	59.3	ref.	–	–	ref.	–	–
Moderate adherence	58	63.7	1.21	0.673	(0.50–2.90)	0.82	0.699	(0.29–2.28)
High adherence	15	71.4	1.72	0.384	(0.51–5.82)	1.26	0.738	(0.33–4.86)
Highest degree in school completed								
No school at all	12	60.0	ref.	–	–	ref.	–	–
Primary	32	66.7	1.33	0.601	(0.45–3.92)	2.20	0.198	(0.66–7.32)
Secondary	49	61.3	1.05	0.918	(0.39–2.87)	1.62	0.405	(0.52–5.00)
Tertiary	55	70.5	1.59	0.369	(0.58–4.41)	3.66	0.037	(1.08–12.40)

^a^Patients with an available baseline and endline interview were included, and patients with a BP < 140&90 at baseline who reported no use of antihypertensive medication (*n* = 2), and patients who were referred to the pilot program from LUTH (*n* = 2), and patients with unknown education status (*n* = 6) were excluded from the analysis

*BP* blood pressure, *HT* hypertension, *BMI* body mass index, *DM* diabetes mellitus

### Adherence to medication and lifestyle advice

Of the patients interviewed at endline 52% self-reported low adherence, 24% moderate adherence and 24% high adherence to antihypertensive medication. This distribution did not significantly differ across the pharmacies (Fischer’s exact-test, *p*-value = 0.229). In addition, no difference in self-reported adherence was observed among those with and without activity in the mHealth data beyond enrollment (Chi^2^-test, *p-*value = 0.408). Forty-six percent of patients who were already in care before the pilot program (*n* = 155) self-reported improved medication adherence at endline compared to baseline. Among patients with low adherence at baseline this was 69% (53 out 77 patients). With respect to lifestyle counseling given at the pharmacy: 12% reported low adherence to given advice, 40% reported moderated adherence, 9% reported high adherence, and 39% reported they did not receive advice.

### Quality of care

A median lag time of two days (IQR: 1–5) was observed in the mHealth data between the blood pressure measurement recorded by the pharmacists and the cardiologist’s response during the pilot program. The protocol called for cardiologists to respond within five days; in 89 of the 417 recorded consultations (21%) the response was late. Six patients had in total eight extremely high blood pressure measurements (SBP ≥ 180/DBP ≥ 110), which created an automatic alert in the mHealth app. In these cases, the cardiologist was expected to respond within two days. In four of these eight recorded consultations the cardiologist responded within two days (min.: 0, max.: 6).

Of the 236 patients with an endline interview, 37 (16%) described experiencing side effects or complaints from their antihypertensive medication during the pilot program. The side effects or complaints frequently reported were frequent urination, fatigue, headache, drowsiness, and dizziness. Seventy-three percent (27 of 37) of patients reported to visit a healthcare provider for these side effects or complaints. Twenty-four patients (89%) visited the pharmacy, of whom four patients also visited another provider, and three patients visited another provider without informing the pharmacy. Of the patients who visited the pharmacy, 83% reported being happy with the way their complaint or side effects were handled or managed. No patients reported CVD events during the study period, whereas six patients reported developing a chronic disease (two of them Diabetes Mellitus, and other non-CVD related).

### Satisfaction with the pilot program

The explanations given by the pharmacists for the discrepancies between the mHealth data and patient’s self-reported data were the use of paper records alongside the app, shortage of staff, and the app not being user-friendly. Most pharmacists and cardiologists explained they had to get used to the new technology. Struggles with the mHealth app, especially at the beginning of the pilot program, such as connectivity, communication between pharmacists and cardiologists, entering passwords, and browsing needed to be overcome. “*We have to look for a way to do it without necessarily having internet, because we live in a country where the best internet provider can’t guarantee you internet service 24/7*” (IDI cardiologist). Moreover, pharmacists explained to pay the pilot program fee for some patients who refused to present as pilot program participants. This because patients did not want to pay the pilot program fee for hypertension care that is normally provided for free by the pharmacy: “*Some paid. Some didn’t. I thought I would rather add some money than start dragging them [patients] for money. So, when OMRON was asking me for data, I was like let’s leave data out of this*” (IDI pharmacist). Pharmacists valued the mHealth app in improving their administration facilitating good blood pressure monitoring and keeping an eye on non-adherent patients.

Pharmacists were content with the care model, because they regarded it as increasing patients’ access to hypertension care: “*Can you compare it? Walking to the pharmacy whenever it is convenient or spending the whole day at LUTH*” (FGD pharmacists). Likewise, most patients explained during IDIs and FGDs being content with the pilot program as they could avoid the hospital. However, they were often not aware of the role of the mHealth app and the cardiologists and did not consider hypertension care at the pharmacy have changed during the pilot program. One patient explained: “*After they [cardiologists] gave me that prescription and test, I just walked away. They don’t know what became of me. So maybe a follow up is nice*” (IDI patient). Patients recommended presence of a medical doctor at times in the pharmacies. Patients were satisfied with the pharmacist monitoring their blood pressure and were of the opinion they gave good advice. Furthermore, they appreciated the good relationship with their pharmacist, the personal care, and accessibility of the pharmacy, saving them time and money. Some patients explained the pilot program helped them to take their hypertension more seriously.

The cardiologists approved the care model, mainly as they realized there is a need for task-shifting to the pharmacies as clinics were understaffed and packed with patients: “*This is the future, clinics are too busy, the app is good*” (IDI cardiologist). Cardiologists opinioned that the pilot program improved quality of hypertension care and bridged the gap between pharmacists and cardiologists. Likewise, the pharmacists appreciated the feedback from the cardiologists enabling them to deliver quality care. Nevertheless, some cardiologists expressed worries about pharmacists taking over the medical doctor’s role and some patients’ inability to differentiate between pharmacists and medical doctors. Most pharmacists explained staff shortages complicated good implementation of the pilot program. The pilot program increased workload, because of reminding patients to attend the pharmacy for care and control. One pharmacy hired an extra staff to run the pilot program. Cardiologists referred to the extended working hours as pharmacists could contact them at odd hours.

Pharmacists explained other barriers, such as patients feeling too strictly monitored, not (always) adhering to treatment, or reluctance to pay. Pharmacists underlined the impact of the recession and their patients’ inability to pay for care or sometimes choosing cheaper healthcare providers, such as chemists. Although patients mentioned the pilot program fee was not expensive, half of the patients reported they faced financial constraints hindering payment of the pilot program fee and/or antihypertensive medication. Furthermore, many patients referred to financial advantages of the pilot program, such as the ability to postpone payment for their drugs at times or get a discount. They stressed that fewer medication stock-outs occurred.

## Discussion

This study was part of a larger study investigating the feasibility of a pharmacy-based hypertension care model employing an mHealth app for remote patient monitoring by cardiologists in Lagos [18, 27]. Pharmacies constituted a beneficial care provider for patients due to accessibility, attention, adherence, and information provision and we observed that patients’ blood pressure reduced. In addition, pharmacists and cardiologists valued the pilot program because of task-shifting, the involvement of cardiologists safeguarding quality of care, and in assisting pharmacists to monitor patients’ adherence. Based on these outcomes we consider the concept of the pilot program feasible and implementable. Areas for improvement are the usability of the mHealth app, pharmacy characteristics and responsibilities, increased visibility of the cardiologists for patients, and the design of the financing model.

The implementation of mHealth within the care model requires improvement. Triangulation of our data sources suggests that the mHealth data did not reflect all care that patients received as part of the pilot program: patients self-reported more visits compared to the mHealth data, and a substantial number of patients (84%) who, according to mHealth data were inactive after enrollment, self-reported visits during the program. The lack of association between decreased blood pressure and retention in the mHealth app, and the feedback from the pharmacists during exit interviews confirm these findings. We assumed that the cardiologists managed patients according to national guidelines, and that the pharmacies supplied good quality medications, and thus would not have caused the discrepancies. Patients continued their hypertension care at the pharmacy without being recorded in the mHealth app, preventing cardiologist from remotely monitoring patients, and thus compromising quality of care. The non-recording of pharmacy visits in the mHealth app contributed to a lack of association between retention in care and blood pressure, although we also observed no association with self-reported retention. Meaning that how retention was measured did not reflect the care patients received or the follow-up duration of the pilot program was too short. As further detailed in our other report [18], healthcare providers and patients perceived mHealth as being attractive, but they also raised concerns on the stability of internet connections and confusion around new technologies. Factors to be addressed include the usability and feasibility of the mHealth app, both recognized as critical in feasibility studies of other mHealth applications [15]. For example, generating automated messages to remind patients of their visits and medication pick-ups, enhancing the connectivity and log-in options, improving the flow of communication between the cardiologist and pharmacist through the app, provision of continuous training on app usage and data entry, and improving the lay-out of the interface. Furthermore, the monthly patient fee interfered with the use of the mHealth app and appeared difficult to sustain in this setting where hypertension care without the mHealth app was provided before and alongside the pilot program. The financing model, the costs of implementation and the availability of pharmacists should be a focus of further study. Health insurance providers or other healthcare financers may potentially fill this gap. Also, the benefits from the pharmacists’ perspective could be enhanced by incentives that make it more profitable.

The blood pressure improvement that patients experienced during the study can probably be attributed to the medical review at enrollment (including revision of treatment regimen), increased attention (reminders, phone calls) by the pharmacist, and patients’ improved self-reported adherence to medication. Blood pressure on target increased from 24% at baseline to 56% at endline. We considered this a relevant outcome, given the programmatic conditions of the pilot program in which any improvement in blood pressure is beneficial to patients. However, by recently released US guidelines for the management of hypertension, defining a blood pressure as elevated above 130/80 [32], blood pressure control in this pilot program would be 6% at baseline and 21% at endline.

Blood pressure improvement and retention in the pilot program differed considerably between the pharmacies. Pharmacy characteristics should be further investigated when rolling out such a care model. A trusted patient-pharmacist relationship, and additionally having a consultation room or designated staff for the pilot program featured as enabling factors in our other report on barriers and facilitators [18], but this study was too small to draw conclusions about pharmacy selection. In addition, for further roll-out attention should be paid to lifestyle and medication counselling protocols. Another aspect to improve may be increased visibility of a medical doctor for patients and better clarification of their role in the care model. Whereas task-shifting was valued by the cardiologists, it also created uncertainty as they feared patients had difficulties differentiating between the role of medical doctors and pharmacists [18, 20]. The establishment of clusters of pharmacies and medical doctors monitoring one patient population may be a way forward to create a better interaction between pharmacists, medical doctors, and patients, and to increase accessibility of the medical doctor for the patient if complications arise or for yearly check-ups.

### Strengths and limitations

An important strength of this feasibility study is the use of different data sources: clinical data recorded by healthcare providers through the mHealth app, structured baseline and endline interviews and qualitative data. The qualitative part provided an in-depth understanding of retention in care and satisfaction with the pilot program. We evaluated not only the implementation of the care model but also its health impact, as is recommended for mHealth interventions [33]. Blood pressure measurements were independent from the clinical recordings in the mHealth app. We only assessed patients included in the pilot program and did not include a control group in the design of the study, since the main aim was to investigate the feasibility of the care model. Since it was difficult to recruit patients already in hypertension care at LUTH [27], we did not estimate the costs of pharmacy-based care compared with costs of regular care from a patient perspective. Costs of implementation and possible savings achieved by task-shifting from medical doctors to the pharmacy staff were also not estimated.

Patients’ retention in care was challenging to measure. In our preparations we overestimated the quality of data collected through the mHealth app and underestimated technical problems, necessitating revision of our definition of retention in care (see Additional file 1). Nevertheless, triangulation of information obtained with different methods provided us with a better understanding of retention in care. By comparing patients’ responses at endline with the mHealth data, we learned that part of the visits as recalled by the patients were not recorded in the mHealth app. After program closure we discussed these discrepancies with the pharmacists in open interviews but did not revisit patients. In future programs, patients’ reasons for dropping-out should be further investigated.

Patients who were lost to follow-up at endline did not differ in age, systolic and diastolic blood pressure at baseline, however, their activity and duration of activity in the mHealth data after enrollment was lower (see Additional file 3). We do not have information on the blood pressure of patients who were lost to follow-up and therefore do not know how this influenced the results of the association with retention in care.

Although pharmacists reported that patients were reluctant to pay the pilot program fee or patient seeking care somewhere else, patients did not mention this during the qualitative research. Socially desirable answers or patient selection may explain some of the patients’ and healthcare providers’ positive attitudes towards the care model.

## Conclusions

Pharmacy-based hypertension care employing mHealth for remote monitoring by cardiologists was shown to be a feasible care model to implement in urban Nigeria, albeit with gaps in the digital data recording. Patients were satisfied with accessibility to, and care given by the pharmacy, whilst the number of patients who had their blood pressure controlled doubled. For further roll-out, the mHealth app usage, pharmacy incentives, and an improved financing model are opportunities to improve the care model. In addition, cost of implementation, the availability of involved healthcare providers needs to be further investigated before such a care model can be implemented in other sub-Sahara African settings.

## Additional files

Additional file 1: Description of the pharmacy-based hypertension care model and sample size calculation. (DOCX 98 kb)

Additional file 2: Sensitivity analyses and second multilevel logistic regression on blood pressure on target and/or improved at endline. (DOCX 20 kb)

Additional file 3: Baseline characteristics of patients stratified by interview at endline. (DOCX 15 kb)

## Abbreviations

CVD: Cardiovascular disease
DBP: Diastolic blood pressure
FGD: Focus group discussion
IDI: In-depth interview
LUTH: Lagos University Teaching Hospital
MMAS-8: 8-item Morisky Medication Adherence Scale
SBP: Systolic blood pressure
SSA: Sub-Sahara Africa
WHO: World Health Organization
