# Supplementary material for: Pharmacy-based hypertension care employing mHealth in Lagos, Nigeria – a mixed methods feasibility study
Source: BMC Health Serv Res. 2018 Dec 4;18:934. doi: 10.1186/s12913-018-3740-3 (PMC6277995; doi:10.1186/s12913-018-3740-3)
Supplement: Supplementary file 1 — Description of the pharmacy-based hypertension care model and sample size calculation. (DOCX 98 kb) [file 12913_2018_3740_MOESM1_ESM.docx]

**Additional file 1.** Description of the pharmacy-based hypertension care model and sample size calculation.

**Care model roles and responsibilities**

The figure shows the communication between the different actors involved in the care model. An elaborate description of the roles of each actor involved in the pilot program is given below the figure.

**Figure.** Description of the care model.

**
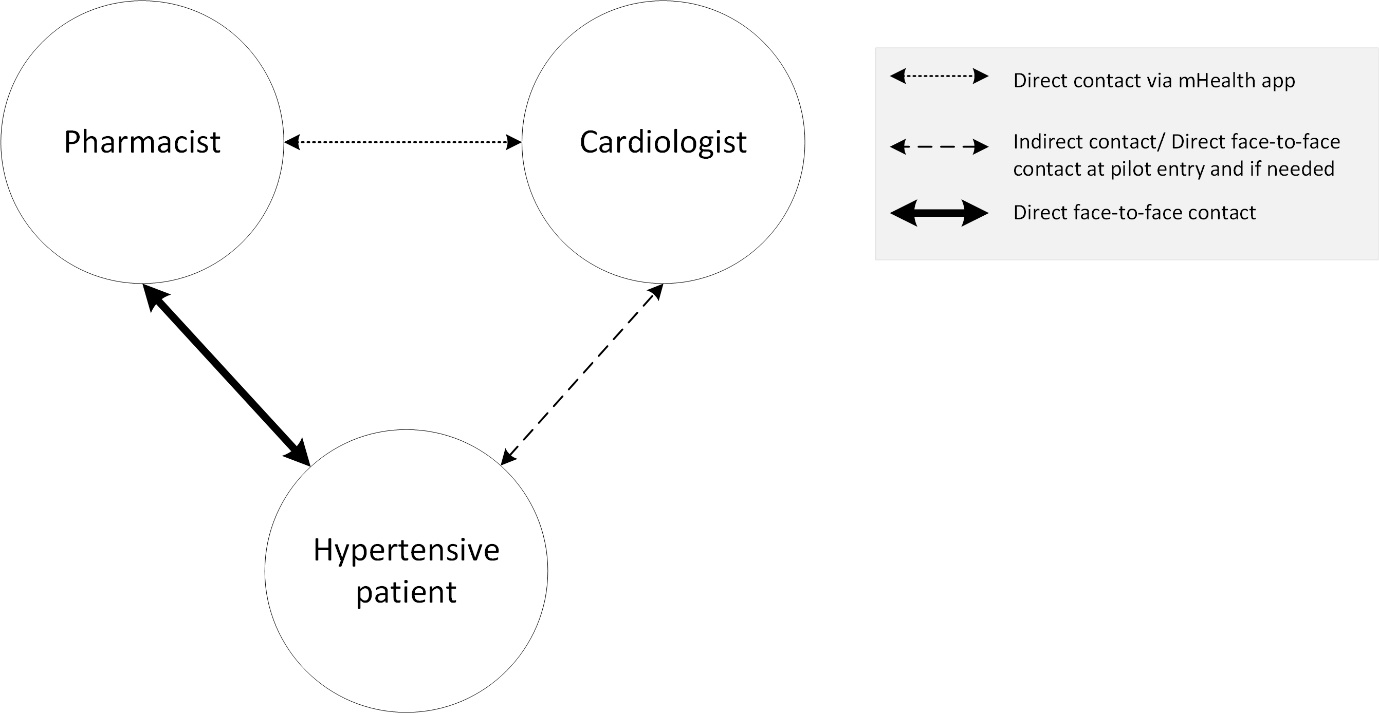
PHARMACIST.** The role of the pharmacy staff was to perform regular consultations with the patients (including blood pressure measurements and medication- and lifestyle counselling), to remind patients of their consultation and outstanding prescriptions, and to communicate with the cardiologist on concerns regarding the patient’s health.

**CARDIOLOGIST.** The role of the cardiologists was to review blood pressure data, possible symptoms or complaints and providing medication prescriptions through a data transfer from the cardiologist to the pharmacy and back. Cardiologists had to respond within 2 or 5 days, depending on the severity of the blood pressure and other signs and symptoms, on data submitted by the pharmacist if the outstanding prescription expired within 12 days. The cardiologists had to call patients in for a face-to-face consultation if they considered it necessary and respond to pharmacist phone calls for urgent assistance when alarm symptoms were present.

**HYPERTENSIVE PATIENT.** Patients were expected to attend scheduled pharmacy and doctor appointments, give an accurate account of their health during consultations, take their antihypertensive medication as prescribed, and pay the patient participation fee of 250 Naira per month (≈0.96USD, average May-Dec 2016) to the pharmacy for the pilot program, this did not include medication costs. After approximately 6 months patients were requested to come to the pharmacy to exit the pilot program, and for referral to regular care.

**Sample size calculation**

In the study protocol, patient retention was defined as the proportion of patients who were included in the program who have attended at least 80% of the drug-pick up visits after six months of follow-up. The pilot program was judged successful by attaining a target of 40% retention. As described in the methods, for this study we used activity in the mHealth app as a proxy for retention. We choose not to adhere to the original definition since the data showed that visits were not recorded in the mHealth app. This was also explained by the pharmacists during our qualitative research.

The sample size of the study was based on the original definition of patient retention. Based on a target of 40% retained after six months of follow-up and a relative precision of 15%, the minimum number of patients needed was 45 per pharmacy. Since five pharmacies participated, our minimum sample size was 225 patients. We also aimed to include an additional stratum of a minimum of 45 patients already receiving antihypertensive care from Lagos University Teaching Hospital (LUTH). The minimum target total sample size was 300 patients, but we aimed to include 500 patients (around 83 per pharmacy and 83 patients already receiving care at LUTH). This sample size also allowed to measure a difference in systolic blood pressure between baseline and six months follow-up of 5 mmHg (standard deviation 20 mmHg) for which a minimum of 169 patients with an endline assessment were needed.
