# Supplementary material for: Pharmacy-based hypertension care employing mHealth in Lagos, Nigeria – a mixed methods feasibility study
Source: BMC Health Serv Res. 2018 Dec 4;18:934. doi: 10.1186/s12913-018-3740-3 (PMC6277995; doi:10.1186/s12913-018-3740-3)
Supplement: Supplementary file 2 — Sensitivity analyses and second multilevel logistic regression on blood pressure on target and/or improved at endline. (DOCX 20 kb) [file 12913_2018_3740_MOESM2_ESM.docx]

**Additional file 2.** Sensitivity analyses and second multilevel logistic regression on blood pressure on target and/or improved at endline.

|  | **Sensitivity analysis 1 (N=226)** | | | | **Sensitivity analysis 2 (N=160)** | | | | | | **Model 2 (N=216)** | | | | | |
| --- | --- | --- | --- | --- | --- | --- | --- | --- | --- | --- | --- | --- | --- | --- | --- | --- |
|  | **OR** | **p-value** | **95% CI** | | **OR** | | **p-value** | | **95% CI** | | **OR** | | **p-value** | | **95% CI** | |
| **Duration active in mHealth app (per month)** | 0.91 | 0.140 | (0.80-1.03) | | 1.07 | | 0.524 | | (0.87-1.30) | | **-** | | **-** | | **-** | |
| **Self-reported at least 6 pharmacy visits** | - | - | - | | - | | - | | - | | 1.55 | | 0.195 | | (0.80-3.02) | |
| **Gender** |  |  |  | |  | |  | |  | |  | |  | |  | |
| Male | ref. | - | - | | ref. | | - | | - | | ref. | | - | | - | |
| Female | 3.82 | 0.001 | (1.70-8.61) | | 2.78 | | 0.059 | | (0.96-8.05) | | 3.65 | | 0.003 | | (1.57-8.46) | |
| **Age at baseline** | 1.05 | 0.001 | (1.02-1.09) | | 1.04 | | 0.067 | | (1.00-1.08) | | 1.04 | | 0.014 | | (1.01-1.07) | |
| **BP classification at baseline** |  |  |  | |  | |  | |  | |  | |  | |  | |
| Stage 1 HT (BP 140-159/90-99) | 0.40 | 0.051 | (0.16-1.00) | | 0.25 | | 0.021 | | (0.08-0.81) | | 0.36 | | 0.040 | | (0.13-0.96) | |
| Stage 2 HT (BP ≥160/100) | 0.21 | 0.002 | (0.08-0.55) | | 0.62 | | 0.451 | | (0.18-2.13) | | 0.54 | | 0.253 | | (0.19-1.55) | |
| Stage 1 HT (BP 140-159/90-99) | ref. | - | - | | ref. | | - | | - | | ref. | | - | | - | |
| **Newly diagnosed** |  |  |  | |  | |  | |  | |  | |  | |  | |
| No | ref. | - | - | | ref. | | - | | - | | ref. | | - | | - | |
| Yes | 0.98 | 0.976 | (0.35-2.76) | | 0.57 | | 0.436 | | (0.14-2.32) | | 1.09 | | 0.877 | | (0.37-3.22) | |
| **On antihypertensive medication at baseline** |  |  |  | |  | |  | |  | |  | |  | |  | |
| No | ref. | - | - | | ref. | | - | | - | | ref. | | - | | - | |
| Yes | 0.56 | 0.213 | (0.22-1.39) | | 0.73 | | 0.599 | | (0.22-2.37) | | 0.68 | | 0.428 | | (0.27-1.75) | |
| **Entry into the pilot program** |  |  |  | |  | |  | |  | |  | |  | |  | |
| Via community screening | ref. | - | - | | ref. | | - | | - | | ref. | | - | | - | |
| Via the pharmacy | 1.42 | 0.328 | (0.71-2.84) | | 1.11 | | 0.811 | | (0.46-2.69) | | 1.35 | | 0.410 | | (0.66-2.76) | |
| **BMI at baseline** | 0.97 | 0.211 | (0.92-1.02) | | 0.98 | | 0.552 | | (0.92-1.05) | | 0.99 | | 0.613 | | (0.93-1.04) | |
| **Self-reported DM** |  |  |  | |  | |  | |  | |  | |  | |  | |
| No | ref. | - | - | | ref. | | - | | - | | ref. | | - | | - | |
| Yes | 0.88 | 0.834 | (0.28-2.80) | | 0.36 | | 0.150 | | (0.09-1.44) | | 0.84 | | 0.775 | | (0.25-2.84) | |
| **Smoking status at baseline** |  |  |  | |  | |  | |  | |  | |  | |  | |
| Not smoking | ref. | - | - | | ref. | | - | | - | | ref. | | - | | - | |
| Quitted | 2.66 | 0.094 | (0.85-8.37) | | 3.94 | | 0.095 | | (0.79-19.69) | | 3.71 | | 0.037 | | (1.09-12.69) | |
| Smokes | 2.09 | 0.552 | (0.18-23.54) | | 0.95 | | 0.971 | | (0.08-11.49) | | 0.79 | | 0.842 | | (0.08-7.89) | |
| **Any alcohol use at baseline** |  |  |  | |  | |  | |  | |  | |  | |  | |
| No | ref. | - | - | | ref. | | - | | - | | ref. | | - | | - | |
| Yes | 0.89 | 0.812 | (0.36-2.24) | | 0.61 | | 0.412 | | (0.18-2.00) | | 0.79 | | 0.633 | | (0.31-2.05) | |
| **Adherence to antihypertensive medication at endline** |  |  |  | |  | |  | |  | |  | |  | |  | |
| Low adherence | ref. | - | - | | ref. | | - | | - | | ref. | | - | | - | |
| Moderate adherence | 1.44 | 0.355 | (0.67-3.09) | | 1.97 | | 0.197 | | (0.70-5.55) | | 1.67 | | 0.223 | | (0.73-3.81) | |
| High adherence | 2.47 | 0.030 | (1.09-5.60) | | 2.48 | | 0.083 | | (0.89-6.94) | | 2.03 | | 0.123 | | (0.83-4.98) | |
| **Adherence to lifestyle advice at endline** |  |  |  | |  | |  | |  | |  | |  | |  | |
| No lifestyle advice given | 2.28 | 0.121 | (0.80-6.47) | | 1.24 | | 0.748 | | (0.34-4.58) | | 1.53 | | 0.428 | | (0.54-4.34) | |
| Low adherence | ref. | - | - | | ref. | | - | | - | | ref. | | - | | - | |
| Moderate adherence | 1.23 | 0.696 | (0.43-3.52) | | 0.81 | | 0.755 | | (0.21-3.08) | | 0.78 | | 0.647 | | (0.27-2.26) | |
| High adherence | 0.98 | 0.976 | (0.25-3.85) | | 3.36 | | 0.232 | | (0.46-24.62) | | 1.96 | | 0.388 | | (0.43-8.97) | |
| **Highest degree in school completed** |  |  |  | |  | |  | |  | |  | |  | |  | |
| No school at all | ref. | - | - | | ref. | | - | | - | | ref. | | - | | - | |
| Primary | 2.04 | 0.261 | (0.59-7.09) | | 2.52 | | 0.249 | | (0.52-12.06) | | 2.41 | | 0.165 | | (0.70-8.32) | |
| Secondary | 1.84 | 0.319 | (0.55-6.15) | | 0.99 | | 0.991 | | (0.23-4.22) | | 1.62 | | 0.416 | | (0.51-5.21) | |
| Tertiary | 4.76 | 0.019 | (1.29-17.48) | | 3.22 | | 0.138 | | (0.69-15.10) | | 4.25 | | 0.023 | | (1.22-14.79) | |
| Sensitivity analysis 1: outcome variable more conservative, same sample as model 1. | | | |  | |  | |  | |  | |  | |  | |  |
| Sensitivity analysis 2: model 1, only for those with activity in the mHealth data beyond baseline. | | | | | | | |  | |  | |  | |  | |  |
| Model 2: Main exposure changed to self-reported pharmacy visits. 10 individuals did not know the number of pharmacy visits they made during the pilot program and are excluded from the analysis | | | | | | | | | | | | | | | |  |
| BP: blood pressure; HT: hypertension; BMI: body mass index; DM: diabetes mellitus | | | | | | | | | | | | | | | |  |
