# Supplementary material for: Pharmacy-based hypertension care employing mHealth in Lagos, Nigeria – a mixed methods feasibility study
Source: BMC Health Serv Res. 2018 Dec 4;18:934. doi: 10.1186/s12913-018-3740-3 (PMC6277995; doi:10.1186/s12913-018-3740-3)
Supplement: Supplementary file 3 — Baseline characteristics of patients stratified by interview at endline. (DOCX 15 kb) [file 12913_2018_3740_MOESM3_ESM.docx]

**Additional file 3.** Baseline characteristics of patients stratified by interview at endline.

|  | **All  (N=328)** | | **Interviewed at endline (N=236)** | | **Not interviewed at endline (N=92)** | | p-value |
| --- | --- | --- | --- | --- | --- | --- | --- |
|  | *n/ mean* | *%/SD/IQR* | *n/ mean* | *%/SD/IQR* | *n/ mean* | *%/SD/IQR* |  |
| **Gender, n (%)** |  |  |  |  |  |  | 0.014 |
| Male | 135 | (41.2) | 107 | (45.3) | 28 | (30.4) |  |
| Female | 193 | (58.8) | 129 | (54.7) | 64 | (69.6) |  |
| **Age, mean (SD)** | 54.9 | (11.9) | 55.1 | (12.1) | 54.5 | (11.5) | 0.688 |
| **Highest degree in school completed, n (%)** |  |  |  |  |  |  | 0.184 |
| No school at all | 37 | (11.3) | 21 | (8.9) | 16 | (17.4) |  |
| Primary | 66 | (20.1) | 48 | (20.3) | 18 | (19.6) |  |
| Secondary | 115 | (35.1) | 85 | (36) | 30 | (32.6) |  |
| Tertiary | 110 | (33.5) | 82 | (34.7) | 28 | (30.4) |  |
| **Systolic BP, mean (SD)** | 147.8 | (16.4) | 147.0 | (15.2) | 149.7 | (19.2) | 0.181 |
| **Diastolic BP, mean (SD)** | 90.9 | (11.4) | 90.4 | (11.3) | 92.2 | (11.7) | 0.204 |
| **BP classification, n (%)** |  |  |  |  |  |  | 0.400 |
| Pre-hypertensive (BP 120-139/80-89) | 2 | (0.6) | 2 | (0.8) | 0 | (0) |  |
| Stage 1 HT (BP 140-159/90-99) | 142 | (43.3) | 105 | (44.5) | 37 | (40.2) |  |
| Stage 2 HT (BP ≥160/100) | 107 | (32.6) | 71 | (30.1) | 36 | (39.1) |  |
| BP on target | 77 | (23.5) | 58 | (24.6) | 19 | (20.7) |  |
| **Newly diagnosed, n (%)** | 65 | (19.8) | 43 | (18.2) | 22 | (23.9) | 0.245 |
| **On antihypertensive medication, n (%)** | 212 | (64.6) | 157 | (66.5) | 55 | (59.8) | 0.251 |
| **Entry into the pilot program, n (%)** |  |  |  |  |  |  | 0.659 |
| Via community screening | 100 | (30.5) | 69 | (29.2) | 31 | (33.7) |  |
| Via pharmacy | 226 | (68.9) | 165 | (69.9) | 61 | (66.3) |  |
| Via LUTH | 2 | (0.6) | 2 | (0.8) | 0 | (0) |  |
| **BMI, mean (SD)** | 28.6 | (6.1) | 28.6 | (6.2) | 28.9 | (5.9) | 0.685 |
| **Self-reported DM, n (%)** | 29 | (8.8) | 19 | (8.1) | 10 | (10.9) | 0.419 |
| **Smoking status, n (%)** |  |  |  |  |  |  | 0.535 |
| Not smoking | 285 | (86.9) | 207 | (87.7) | 78 | (84.8) |  |
| Quitted | 36 | (11) | 25 | (10.6) | 11 | (12) |  |
| Smokes | 7 | (2.1) | 4 | (1.7) | 3 | (3.3) |  |
| **Any alcohol use, n (%)** | 97 | (29.6) | 68 | (28.8) | 29 | (31.5) | 0.629 |
| **Activity in mHealth data after enrollment, n (%)** | 220 | (67.1) | 167 | (70.8) | 53 | (57.6) | 0.023 |
| **Activity in mHealth data in months (if active), median (IQR)** | 3.0 | (2.1-5.1) | 3.3 | (2.1-5.4) | 2.3 | (1.8-3.2) | <0.001 |
| BP: blood pressure; HT: hypertension; LUTH: Lagos University Teaching Hospital; BMI: body mass index; DM: diabetes mellitus | | | | | | | |
